# Mirtazapine in Treating Functional Dyspepsia: A Systematic Review and Meta-Analysis

**DOI:** 10.14740/gr2157

**Published:** 2026-08-26

**Authors:** Hao The Nguyen, Arman Vaghefi, Paul Pardo, David Pitts

**Affiliations:** a Department of Internal Medicine, Keesler Medical Center, Biloxi, MS, USA; b Department of Internal Medicine, University of New Mexico, Albuquerque, NM, USA; c College of Arts and Sciences, University of South Florida, Tampa, FL, USA; d Department of Family Medicine, Alexander T Augusta Military Medical Center, Fort Belvoir, VA, USA

**Keywords:** Mirtazapine, Functional dyspepsia, Disease of gut–brain interaction

## Abstract

**Background:**

Functional dyspepsia (FD) is a disease of gut–brain interaction that impacts the quality of life of many patients with limited effective pharmacologic options. Neuromodulators are increasingly used for refractory symptoms, but the efficacy of mirtazapine remains poorly defined. Although multiple randomized trials have evaluated mirtazapine for FD, its effects have not previously been pooled in a focused meta-analysis. The objective of our study was to synthesize results of randomized controlled trials (RCTs) to clarify their efficacy in treating this condition.

**Methods:**

A systematic search of PubMed, Embase, and Cochrane was performed to identify studies evaluating mirtazapine for FD from inception through November 2025 (PROSPERO ID: CRD420251237233). Ninety-three records were identified through databases and two through additional sources. After removal of duplicates and screening of abstracts, six full-text articles were assessed. Three were excluded for lacking an appropriate control group or failing to meet predefined eligibility criteria, while three RCTs were included (n = 154). Study-level effect sizes comparing mirtazapine and control groups at the end of treatment were calculated and pooled using a random-effects model. Heterogeneity was assessed using I^2^. Robustness was examined using leave-one-out analysis and influence diagnostics were visualized with a Baujat plot.

**Results:**

Across three RCTs, mirtazapine was associated with significantly lower FD symptom severity compared with control treatment. The pooled effect size demonstrated a large benefit, with a standardized mean difference of −1.39 (95% confidence interval (CI): −2.00 to −0.78). Heterogeneity was substantial (I^2^ = 65.1%), which likely reflects variation in enrolled populations and dosing. Two trials focused on FD patients with associated weight loss, with one additionally requiring comorbid depressive symptoms. These studies also required washout of other gastrointestinal medications, while the third started continued background treatment. However, the overall pooled effect remained directionally consistent and clinically meaningful across all sensitivity tests, including leave-one-out analysis.

**Conclusion:**

Mirtazapine was associated with significantly lower symptom severity compared with control treatment across available RCTs of FD. Increased heterogeneity may reflect differences in dosing, patient selection, and study design. Despite this, sensitivity analyses indicate that the observed effect is robust. Given the limited therapeutic options for refractory FD, mirtazapine may warrant consideration in select patient populations. However, larger and more methodologically uniform trials are needed to better define its role before considering clinical use.

## Introduction

Functional dyspepsia (FD) is a highly prevalent disorder of gut–brain interaction characterized by chronic upper gastrointestinal (GI) symptoms, including postprandial fullness, early satiety, epigastric pain, and burning, in the absence of structural disease. It affects an estimated 10–20% of the global population and is associated with substantial impairment in quality of life, increased healthcare utilization, and significant socioeconomic burden [1]. Despite its prevalence, the pathophysiology of FD remains incompletely understood and is thought to be multifactorial, involving abnormalities in gastric motility, visceral hypersensitivity, impaired gastric accommodation, mucosal immune activation, and dysregulation of central nervous system processing [2]. This has made FD difficult to treat although dietary intervention, eradication of *H. pylori* infection, proton-pump inhibitors, and cognitive behavioral therapy have shown to be effective [3].

Mirtazapine, a noradrenergic and specific serotonergic antidepressant, has been studied as an option for FD, particularly in patients with prominent symptoms of early satiety, weight loss, or impaired gastric accommodation. Its pharmacologic profile, including antagonism of central presynaptic α2-adrenergic receptors and postsynaptic serotonin receptors, may modulate visceral sensitivity, enhance gastric accommodation, and improve appetite [4]. Randomized controlled trials (RCTs) have been performed to evaluate the efficacy of mirtazapine in FD, with some reporting improvements in symptom severity and quality of life. However, findings across studies have been heterogeneous, and individual trials have been limited by small sample sizes and varying outcome measures.

To date, the overall efficacy of mirtazapine in FD has not been clearly established through a focused synthesis of randomized evidence. Given the growing clinical use of neuromodulators and the need for more effective treatment options in refractory FD, a rigorous evaluation of existing data is warranted. Therefore, the objective of this study was to systematically review and meta-analyze RCTs assessing the efficacy of mirtazapine in patients with FD, with the aim of clarifying its role in symptom management and informing clinical practice.

## Materials and Methods

### Study design and registration

This systematic review and meta-analysis was conducted following the Preferred Reporting Items for Systematic Reviews and Meta-Analyses (PRISMA) guidelines [5]. The study protocol was prospectively registered in the International Prospective Register of Systematic Reviews (CRD420251237233). The objective was to synthesize RCT evidence evaluating the efficacy of mirtazapine for the treatment of FD.

### Search strategy

A comprehensive literature search was performed using PubMed, Embase, and the Cochrane Central Register of Controlled Trials (CENTRAL) from database inception through November 2025. Search terms included combinations of controlled vocabulary and keywords related to “mirtazapine” and “functional dyspepsia.” Reference lists of relevant studies and prior reviews were manually screened to identify additional eligible studies. No language restrictions were applied during the search.

### Eligibility criteria

Studies were included if they met the following criteria: (1) population: adult participants (≥ 18 years) diagnosed with FD using Rome IV criteria or equivalent; (2) intervention: mirtazapine therapy; (3) outcomes: validated FD symptom scores pre- and post-treatment; (4) design: RCTs.

Studies were excluded if they were non-randomized, observational, or crossover without separable data. Studies focusing on other functional GI disorders without isolated FD data and pediatric populations were also excluded.

### Study selection and data extraction

Titles and abstracts were screened independently by two reviewers, followed by full-text review of potentially eligible studies. Discrepancies were resolved through consensus. Data extraction was performed independently by two reviewers using a standardized form. Extracted variables included study characteristics (author, year, design), sample size, patient demographics, diagnostic criteria, intervention details (dose and duration of mirtazapine), comparator characteristics, and outcome measures. For quantitative synthesis, mean changes in symptom severity scores and corresponding standard deviations were extracted or calculated from reported data.

### Statistical analysis

The primary outcome was global FD symptom severity at the end of treatment, comparing mirtazapine and control groups. Effect sizes were calculated as standardized mean differences (SMDs) with 95% confidence intervals (CIs) using Hedges’ g to account for small sample sizes. Study-level effect sizes comparing mirtazapine and control groups at the end of treatment were calculated using a random-effects model (DerSimonian–Laird method) to pool results. This was decided given the anticipated clinical and methodological heterogeneity across studies. Statistical heterogeneity was assessed using Cochran’s Q test and quantified with the I^2^ statistic, with values above 50% considered indicative of substantial heterogeneity.

Sensitivity analyses were conducted using a leave-one-out approach to evaluate the robustness of pooled estimates. Study-level influence and contribution to heterogeneity were further explored using influence diagnostics and Baujat plots. Forest plots were generated to visualize individual and pooled effect sizes. All statistical analyses were conducted using Python (version 3.11).

## Results

### Study selection

**Figure 1:**
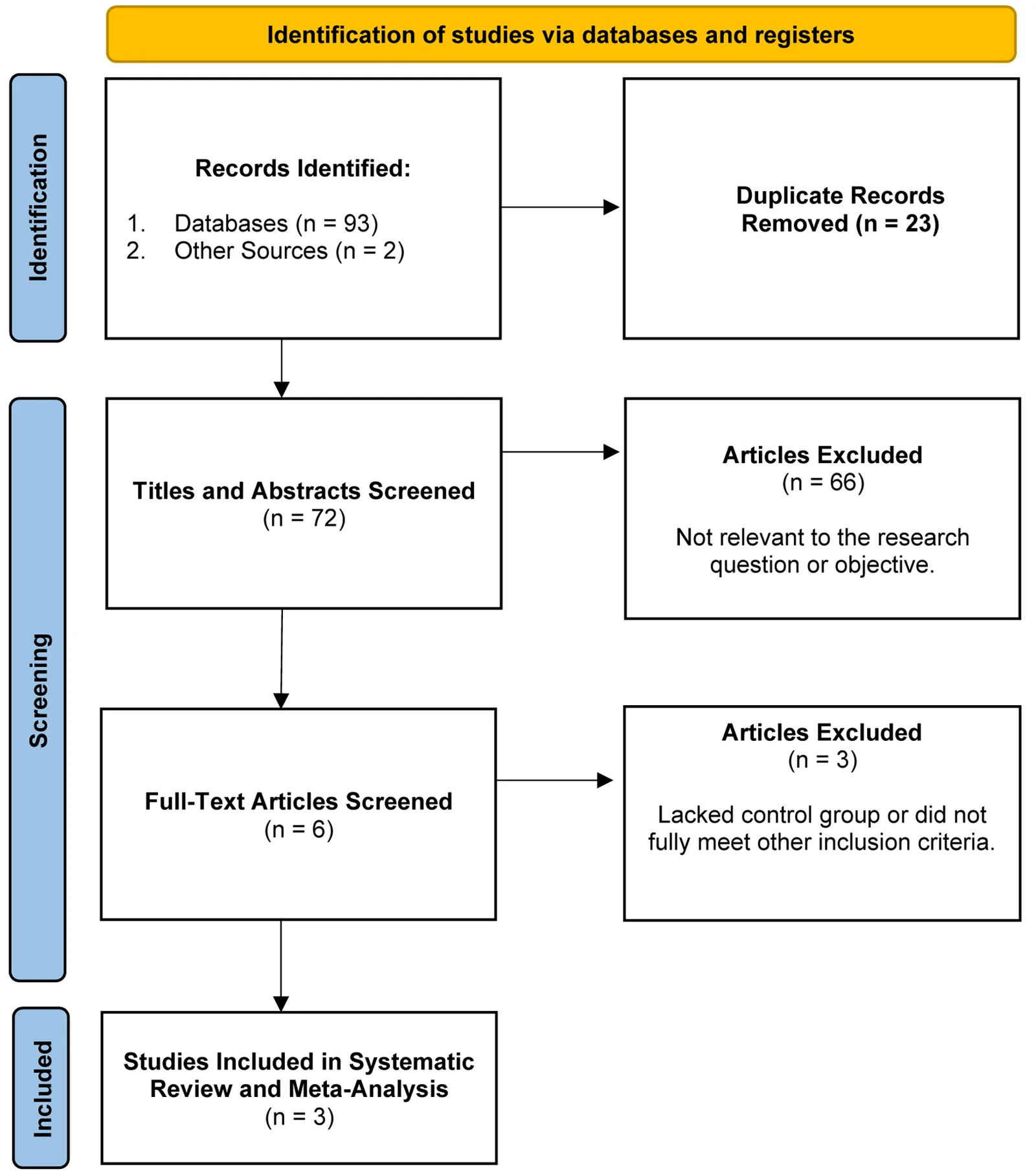
PRISMA diagram outlining the process of selecting studies for meta-analysis.

The literature search identified 95 records (93 from databases and two from additional sources). After removal of 23 duplicates, 72 titles and abstracts were screened, of which 66 were excluded. Six full-text articles were assessed for eligibility, and three studies were excluded due to lack of an appropriate control group or failure to meet pre-defined inclusion criteria. A total of three RCTs comprising 154 patients were included in the final analysis. The selection process is detailed in Figure 1.

### Study characteristics

The included RCTs were published between 2016 and 2025 and evaluated mirtazapine in adult patients with FD over treatment durations of approximately 8 weeks. Sample sizes ranged from 34 to 117 participants. Two studies enrolled patients with FD and associated weight loss, with one additionally requiring comorbid depressive symptoms. The third included a broader FD population. Mirtazapine dosing was either 15 or 30 mg daily. All studies utilized the Nepean Dyspepsia Symptom Index. All three studies demonstrated lower symptom severity in the mirtazapine group compared with control at the end of treatment. Additional study characteristics are summarized in Table 1 [6–8].

**Table 1:** Patient Population, Intervention, and Other Key Characteristics of Included Studies in Our Meta-Analysis

Study	Population	Treatment group, n	Control group, n	Sex	Age	Duration	Dose	Other treatments	Symptom scale	Effect size (95% CI)
Jiang 2016 [6]	Adults with weight loss and diagnosis of depression and FD	20	20	Treatment group: 8M/12F Control group: 7M/13F	Treatment group: 43.45 ± 11.50 Control group: 39.95 ± 6.84	8 weeks	30 mg	None	NDSI	−1.08 (−1.73 to −0.43)
Tack, 2016 [7]	Adults with weight loss and FD, without diagnosis of depression or anxiety	17	17	Treatment group: 3M/14F Control group: 2M/15F	Treatment group: 35.9 ± 2.3 Control group: 39.9 ± 3.5	8 weeks	15 mg	None	NDSI	−2.24 (−3.09 to −1.40)
Cao 2025 [8]	Adults with diagnosis of FD	58	59	Treatment group: 29M/29F Control group: 37M/22F	Treatment group: 43.93 ± 13.27 Control group: 43.58 ± 11.86	8 weeks	15 mg	Pancreatic enzyme supplement and domperidone	NDSI	−1.11 (−1.50 to −0.73)

CI: confidence interval; FD: functional dyspepsia; NDSI: Nepean Dyspepsia Symptom Index.

### Pooled analysis

**Figure 2:**
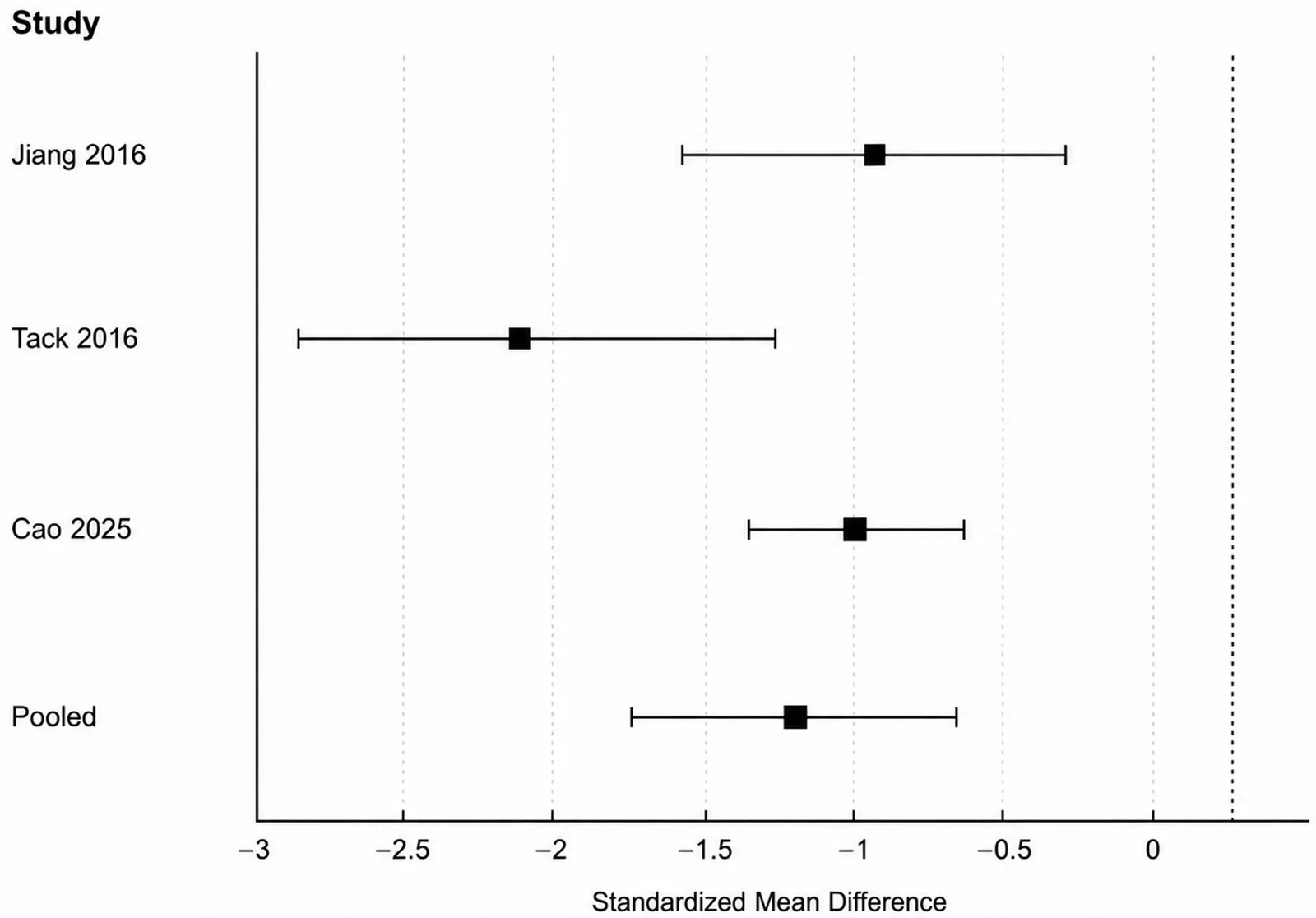
Forest plot of pooled effect sizes comparing mirtazapine and control treatment for functional dyspepsia symptom severity.

**Figure 3:**
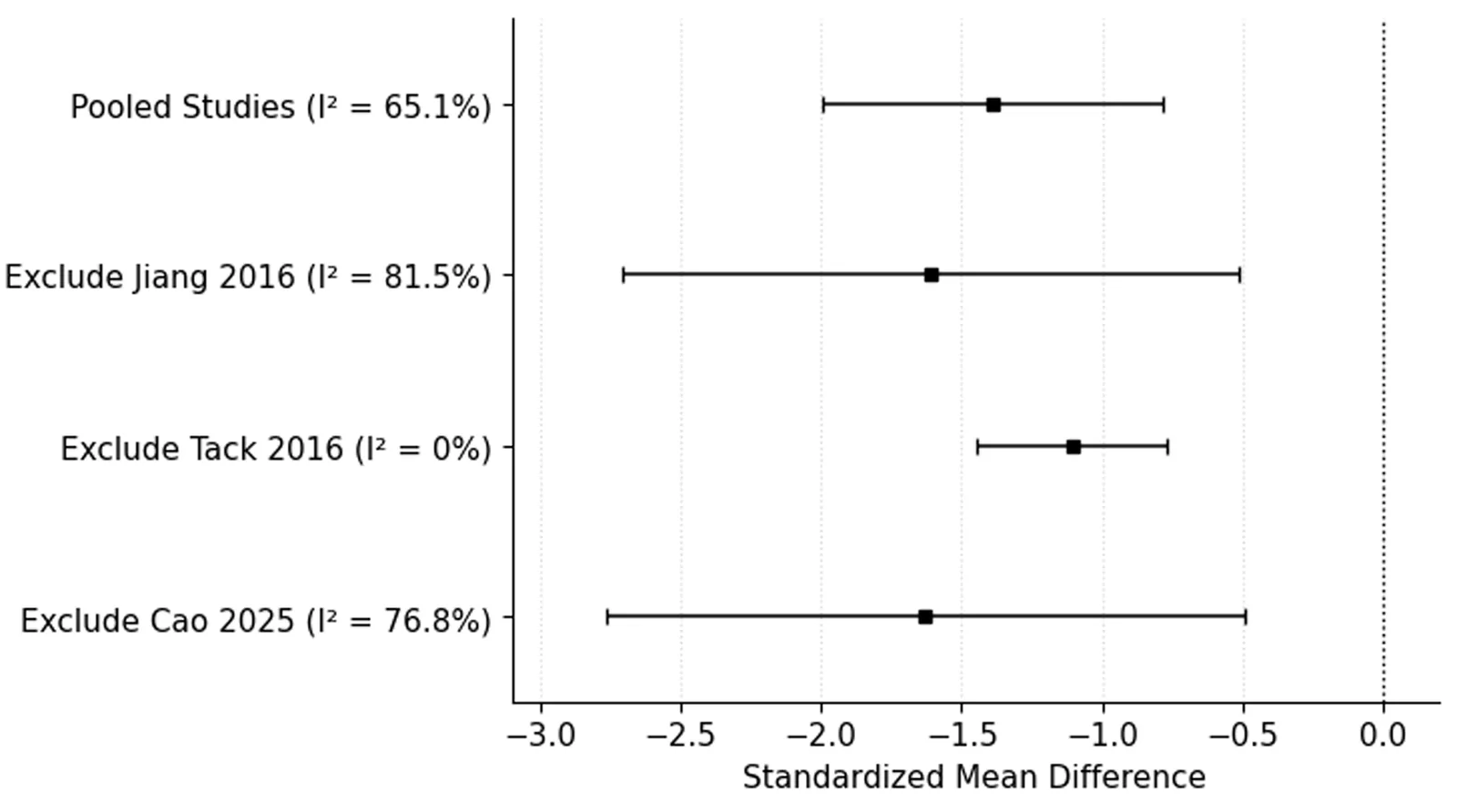
Leave-one-out sensitivity analysis of mirtazapine versus control for functional dyspepsia symptom severity.

**Figure 4:**
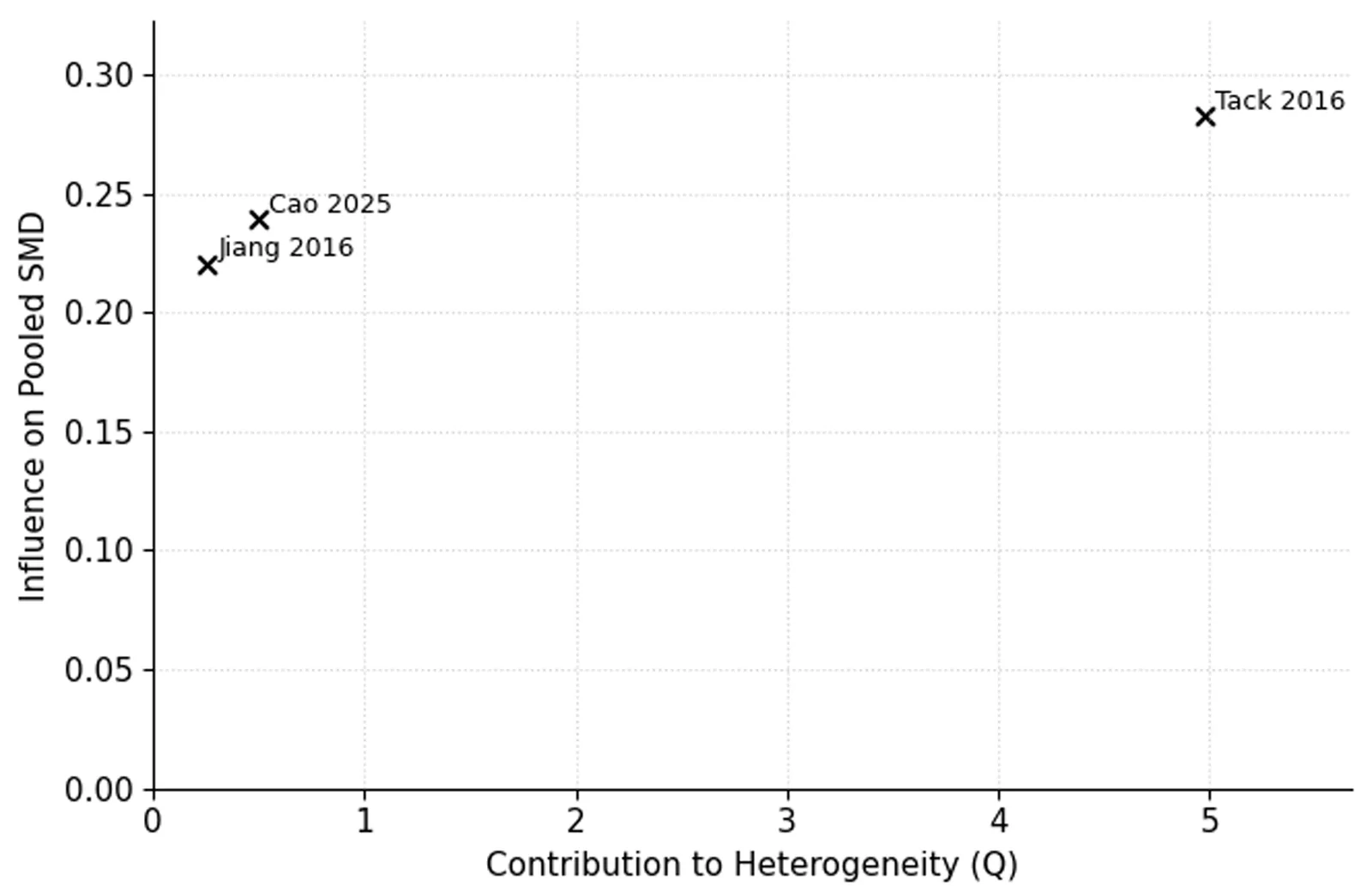
Baujat plot displaying the effect of each study on heterogeneity.

Across three RCTs, mirtazapine was associated with significantly lower FD symptom severity compared with control treatment. The pooled analysis demonstrated an SMD of −1.39 (95% CI: −2.00 to −0.78), indicating significantly lower symptom severity among patients receiving mirtazapine compared with control treatment (Fig. 2). There was substantial heterogeneity among studies (I^2^ = 65.1%), indicating variability in effect sizes across the included trials. Leave-one-out sensitivity analysis demonstrated that the overall estimate remained directionally consistent across all iterations (Fig. 3). Exclusion of individual studies did not alter the direction of effect, though the magnitude of the pooled estimate and degree of heterogeneity varied. Influence diagnostics using a Baujat plot identified Tack et al as the primary contributor to between-study variability (Fig. 4). This was supported with the leave-one-out analysis, as removal of this study resulted in minimal heterogeneity between the remaining studies.

### Publication bias

Formal assessment of publication bias was not performed due to the limited number of included studies.

## Discussion

This analysis suggests that treatment with mirtazapine was associated with significantly lower symptom severity compared with control treatment in patients with FD. The pooled effect demonstrated a large magnitude of improvement, and the direction of effect was consistent across all included studies, although variability in effect size was observed.

Despite the consistency in direction of effect, there was moderate-to-substantial heterogeneity across studies. This variability may reflect differences in study populations, including the inclusion of patients with weight loss or comorbid depressive symptoms, as well as differences in study design, baseline symptom severity, and treatment protocols [6, 7]. Variability in concomitant therapies and inclusion criteria across trials may also have contributed to differences in observed effect sizes [8]. Sensitivity analyses demonstrated that the overall findings were robust, although individual studies contributed differently to heterogeneity. Exclusion of Tack et al reduced the heterogeneity from 65.1% to minimal levels, suggesting that this study was the primary driver in between-study variability. This may be because their patient population excluded patients with a concurrent diagnosis of depression or anxiety, which are known risk factors for FD [9]. This is relevant as previous publications have commented on mirtazapine’s antagonistic effects on histamine receptors, a2 adrenergic receptors, 5-HT2C, and 5-HT3 receptors improving dyspeptic symptoms although use has been limited to treatment of depressive and anxiety disorders [8]. These patient populations may be among the subgroups that could benefit the most with concurrent FD.

Several limitations should be considered when interpreting these findings. First, the number of included studies was small, limiting the ability to perform subgroup analyses or formally assess publication bias. Second, the included studies differed in patient selection criteria, dosing strategies, and background treatment protocols, which may affect generalizability and contribute to observed heterogeneity. Additionally, outcome data from some studies required extraction from published figures because complete numerical datasets were not available. Despite these limitations, all included studies demonstrated treatment effects favoring mirtazapine, and the overall findings remained stable across sensitivity analyses. These differences could represent clinically distinct FD phenotypes and treatment contexts, which likely contributed to the significance in heterogeneity (I^2^ = 65.1) in this study [10].

### Conclusion

In this systematic review and meta-analysis, mirtazapine was associated with significantly lower symptom severity compared with control treatment across available RCTs of FD. Despite substantial heterogeneity across studies, the direction of effect remained stable across sensitivity analyses, supporting the robustness of the observed findings. These results suggest that mirtazapine may be associated with meaningful symptomatic improvement in patients with FD, particularly in populations represented within the included trials.

The findings of this study are limited by differences in patient population and modest sample sizes. This study does show that there is potential for mirtazapine to be considered as a second-line agent for pharmacologic therapy of FD, alongside other neuromodulators such as tricyclic antidepressants. Further large, well-designed RCTs with standardized methodologies and outcome measures are needed to better define the clinical role of mirtazapine in FD and to clarify its therapeutic effect relative to existing treatment options.

## Data Availability

All data analyzed during this study were extracted from published articles. Additional data and materials are available from the corresponding author upon reasonable request.

## Abbreviations

FD: functional dyspepsia
GI: gastrointestinal
PRISMA: Preferred Reporting Items for Systematic Reviews and Meta-Analysis
RCT: randomized controlled trial
SMD: standardized mean difference
